# Plasma exosome-derived fragile site-associated tumor suppressor as a powerful prognostic predictor for patients with ovarian cancer

**DOI:** 10.17305/bjbms.2021.6404

**Published:** 2021-09-07

**Authors:** Renjing Hu, Xiaochun Chen, Shiliang Zhang, Bin Liu, Hao Pei, Fan Tu, Jun Liu, Hao Yu

**Affiliations:** 1 Department of Laboratory Medicine, Wuxi Second People’s Hospital, Jiangsu, China; 2 Department of Laboratory Medicine, Taizhou Second People’s Hospital, Taizhou, Jiangsu, China; 3 Department of Laboratory Medicine, Wuxi Fifth People’s Hospital, Jiangsu, China; 4 Department of Interventional Oncology, Wuxi Fifth People’s Hospital, Jiangsu, China

**Keywords:** Fragile site-associated tumor suppressor, ovarian cancer, exosomes, prognosis, predictive value

## Abstract

The objective of the study was to investigate the levels of plasma exosome-derived fragile site-associated tumor suppressor (FATS) and evaluate its prognostic predictive ability in ovarian cancer (OC) patients. Exosome-rich fractions were isolated from the plasma of 90 patients with OC enrolled in this study. The levels of plasma exosome-derived FATS were detected by ELISA. The levels of exosome-derived FATS in OC patients were significantly lower as compared to the healthy controls (P < 0.001). The levels of plasma exosome-derived FATS were higher in OC patients with low grade (1/2), and Federation International of Gynecology and Obstetrics (FIGO) Stages I/II than those in high grade (3/4) and Stages III/IV of the disease (*p* = 0.003; *p* < 0.001), respectively. The levels of plasma exosome-derived FATS were significantly higher in OC patients with no lymph node metastasis or no ascites as compared to those with lymph node metastasis or ascites, respectively (both *p* < 0.001). The levels of plasma exosome-derived FATS were higher in OC patients having CA-125 below 35 U/ml as compared to those with CA-125 greater than 35 U/ml (*p* < 0.001). Among all enrolled OC patients, both 5-DFS and 5-OS were shorter in patients with lower plasma exosome-derived FATS levels than those with higher levels (both *p* < 0.001). The area under the receiver operating characteristic curve of plasma exosome-derived FATS was 0.85 (95% CI: 0.76-0.91) for 5-DFS and 0.91 (95% CI: 0.83-0.96) for 5-OS prediction in patients with OC. Plasma exosome-derived FATS levels in OC patients were significantly downregulated. Low levels of plasma exosome-derived FATS had a significant relationship with FIGO Stages III/IV, high grade, ascites, higher levels of CA-125, lymph node metastasis, and prognosis of OC patients. Thus, our findings may provide insights for the development of a new strategy OC treatment.

## INTRODUCTION

Ovarian cancer (OC) is a gynecological malignancy in the female reproductive system with the highest fatality rate [1,2]. The lack of obvious pathological features, difficulty in early diagnoses, and lack of accurate tumor markers contribute to its poor prognosis [3]. At present, the incidence of OC is increasing worldwide with the affected population getting younger. In 2018, globally, there were more than 290,000 new cases and approximately 185,000 OC-related deaths [4,5]. At present, the main treatment methods include radical OC surgery, cytoreductive surgery, and platinum combined with paclitaxel chemotherapy; however, the 5-year survival rate remains poor [6].

Oncology research includes the discovery of novel tumor suppressor genes and investigations of their roles in the development of tumors [7]. Common fragile sites (CFSs) are site-specific unstable regions in the normal genome and include the rare fragile sites [8]. While the role of rare fragile sites in tumors remains unclear, CFS is consistent for many tumor genome mutations, and they are closely related to the occurrence and development of tumors [9]. The introns and expression regulatory regions of fragile site-associated tumor suppressor (FATS) gene are rich in AT repeat sequences and have characteristics of typical fragile locus genes [10]. FATS is a tumor suppressor gene associated with DNA damage-induced tumors. Fluorescence in situ hybridization confirmed that FATS is located on the 10q26.2 chromosome fragile site FRA10F. Due to the heterozygous loss in many genes at this locus, FATS may be closely related to the occurrence of human tumors [11]. Furthermore, not only have significant deletions been reported in the FATS gene in breast cancer, OC, and lung cancer patients, but RT-PCR detection also shows that the expression levels of FATS mRNA were either low or absent in OC cells and tumor tissues as compared to the corresponding normal cells and adjacent tissues, respectively. After cotransfection of the FATS expression vector, both *in vitro* and *in vivo* anti-tumor activities have been reported [12,13].

Exosomes are extracellular vesicles secreted by various cells. Their diameter is between 30 and 150 nm. They contain mRNA, proteins, lipids, and other substances. These vesicles can transmit information and substances between cells, and thus, participate in the regulation of cell differentiation and tissue development [14,15]. Several studies report four forms of information exchanges between exosomes and cells as follows: (1) Exosomes act as signal complexes to stimulate target cells; (2) they can transmit receptors between cells; (3) they can deliver functional proteins to recipient cells; and (4) they can transmit genetic information to recipient cells. All these exchanges play a key role in the occurrence, development, invasion, and metastases of tumors [16-18]. Recently, a growing body of evidence has confirmed the important biological regulatory roles of exosomes in the occurrence and development of OC [19,20]. However, there are only a few reports on the diagnostic and prognostic values of exosomes in OC patients. Hence, we aimed to investigate the levels of plasma exosome-derived FATS and assess its prognostic predictive ability for OC patients.

## MATERIALS AND METHODS

### Patients

**FIGURE 1:**
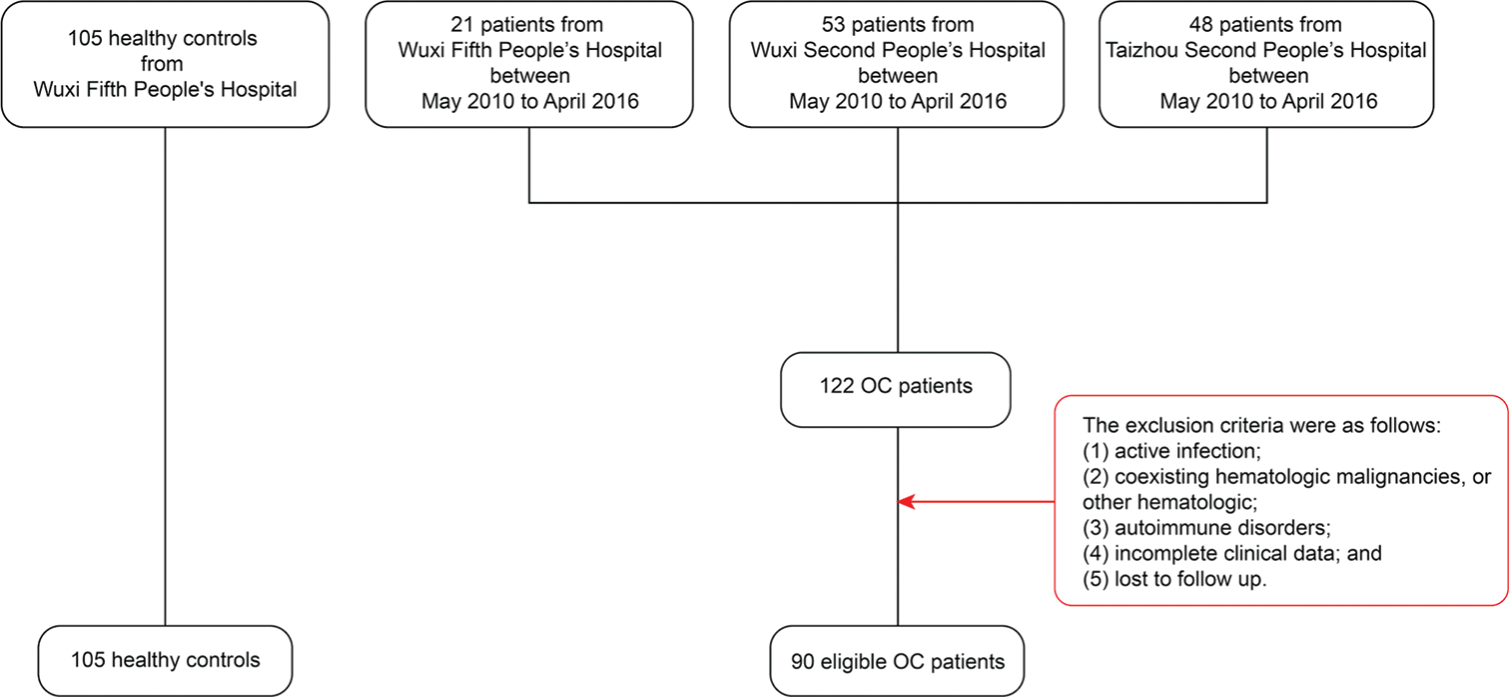
Flowchart depicting ovarian cancer patient enrollment in the three hospitals.

The blood samples of 90 enrolled OC patients were collected from the Second People’s Hospital of Wuxi city, the Fifth People’s Hospital of Wuxi city, and the Second People’s Hospital of Taizhou city between May 2010 and April 2016. Figure 1 shows a flowchart for OC patient enrollment from the three hospitals. Some patients were excluded due to the following: (1) Active infection; (2) coexisting hematological malignancies or other hematological conditions; (3) autoimmune disorders; (4) lack of clinical data; and (5) follow-up failure.

We collected the corresponding clinical data of the 90 OC patients from their medical records. These included demographic characteristics, tumor size, lymph node metastasis, Federation International of Gynecology and Obstetrics (FIGO) stage, and pathological differentiation. The median follow-up duration was 77.5 months (range: 48.0-112.0 months). During the same period, blood samples from 105 healthy controls from the Wuxi Fifth People’s Hospital, aged between 51 and 73 years, with a median age of 65 years old, were also collected. The study design was approved by the hospital’s Ethics Committee (No. 2017003). Informed consent was obtained from each patient.

### Plasma exosome isolation

A 1-2 ml of human plasma was extracted from the cells, diluted 5 times in 1×PBS and then centrifuged at 500×g for 5 minutes at 4°C. After balancing, the supernatant was centrifuged. The temperature of the centrifuge was set at 4°C and centrifugation was performed at 2000 g for 15 minutes. The supernatant was then transferred into a 15 ml centrifuge tube and centrifuged at 4°C. After balancing, the supernatant was collected after centrifugation at 15000 g for 1 hour and then filtered using a 0.22 mm filter. After aseptic filtration, the filtrate was put into a new ultracentrifugation tube. The temperature was set at 4°C and it was centrifuged at 100,000 g for 2 hours. After centrifugation, the supernatant was gently aspirated, leaving behind the precipitate. A 100-200 μl 1×PBS buffer was used to resuspend the exosomes, and these were stored at −80°C until further use.

### Transmission electron microscopy (TEM)

The extracted exosomes stored at −80°C were thawed for further preparation. The extracted exosomes were fixed in 50-100 μl of 2% paraformaldehyde solution. A 5-10 μl of the exosome solution was added to the Formvar carbon sample carrier. A 100 μl PBS was added to the sealing film and the copper mesh was placed on the PBS droplet for cleaning with tweezers. It was incubated with 50 μl of 1% glutaraldehyde for 5 minutes and washed 8 times with 100 μl ddH_2_O for 2 minutes each. It was then incubated with 50 μl of uranium peroxide oxalate (pH 7.0) for 5 minutes, followed by 50 μl methylcellulose solution for 10 minutes. A stainless steel ring was placed on the top of the sample table and the excess liquid was absorbed using a filter paper. The sample was air-dried for 5-10 minutes. The shape of exosomes was observed under the electron microscope. Images were captured.

### Nanoparticle tracking analysis (NTA)

The sample was diluted with water for a final particle concentration of 1×10^7^/ml-1×10^9^/ml. Zeta View PMX110 was used to measure the number and size of particles in the sample using the laser at 405 nm. Thirty photos per second were captured for 1 minute. Based on the detection results, the NTA software (Zeta View 8.02.28) was used to analyze the movement of particles and calculate the number of exosomes.

### Western blotting

The extracted exosomes were diluted with a RIPA lysis buffer to a certain concentration. A 20 ml of the diluted Exo was added to the test sample well in a 96-well plate and incubated at room temperature. The total exosomal proteins were separated by SDS-PAGE. After semi-dry transfer onto PVDF film, the membrane was blocked with 5% skimmed milk and subsequently incubated overnight at 4°C with the respective primary antibodies including Annexin V, TSG101, CD9, and CD63 (Santa Cruz Biotechnology, Inc., Texas, USA). The membrane was incubated at room temperature for 1 hour on a Rocker-Shaker.

### ELISA

The residual cells were removed from the plasma sample and the cell debris was diluted with 1 × PBS (1:500). On the ice, the exosomes were precipitated with 100 ml RIPA lysis buffer for 30 minutes. After shaking and mixing, 1 × PBS (1:3 dilution) was used to dilute the samples. To the ELISA plate coated with FATS antibody (MSKBIO, Wuhan, China), 1 well of blank control and 7 wells of gradient concentration solutions, respectively, were added. The diluted exosome-derived samples had a final concentration of 100 μl. After incubation at 37°C for 1 hour, the liquid in the well was discarded, spun-dried, and then, 100 μl of solution A was added. The membrane was covered and incubated in a 37°C oven for 60 minutes. Subsequently, the plate was washed thrice. A 100 μl of solution B was added, covered with membrane, and incubates in the oven at 37°C for 30 minutes. The plated was washed 5 times. A 90 μl of TMB substrate solution was added, covered with a film, and finally developed in dark at 37°C for 15 minutes. A 50 μl of the termination reaction solution was added. The microplate reader was used to detect the absorbance value at 450 nm.

### Statistical analysis

Statistical analyses were performed using SPSS version 22.0 (IBM, Chicago, IL, USA). The data were shown as mean ± SD. The Chi-square test or Wilcoxon’s rank-sum test was used to perform correlation analyses. ROC curve analysis was used to assess the prognostic predictive ability of plasma exosome-derived FATS. *p* < 0.05 was considered statistically significant.

## RESULTS

### Baseline characteristics

The clinical characteristics of 90 enrolled OC patients are shown in Table 1. Among them, 49 (54.44%) patients were lesser than 60 years of age, and 41 (45.56%) were 60 years of age or older. The CA-125 levels in 11 (12.22%) patients were less than 35 U/ml, while 79 (87.78%) patients had CA-125 levels greater than 35 U/ml. The pathological types included 52 (57.78%) cases of ovarian serous carcinoma, 21 (23.33%) cases of mucinous ovarian carcinoma, and 17 (18.89%) cases of ovarian endometrioid carcinoma. A total of 34 (37.78%) patients were in FIGO Stages I/II, while 56 (62.22%) patients were in FIGO Stages III/IV of the disease. The numbers of patients with low grades (1/2) and high grades (3/4) were 43 (47.78%) and 47 (52.22%), respectively. Tumor size was less than 5 cm in 51 cases (56.67%) and greater than 5 cm in 39 cases (43.33%). In addition, 53 (58.89%) patients had no lymph node metastases, while 37 (41.11%) patients had lymph node metastases. Forty-seven (52.22%) patients had ascites. The tumor position was unilateral among 31(34.44%) patients and bilateral among 59 (65.56%) patients.

**TABLE 1:**
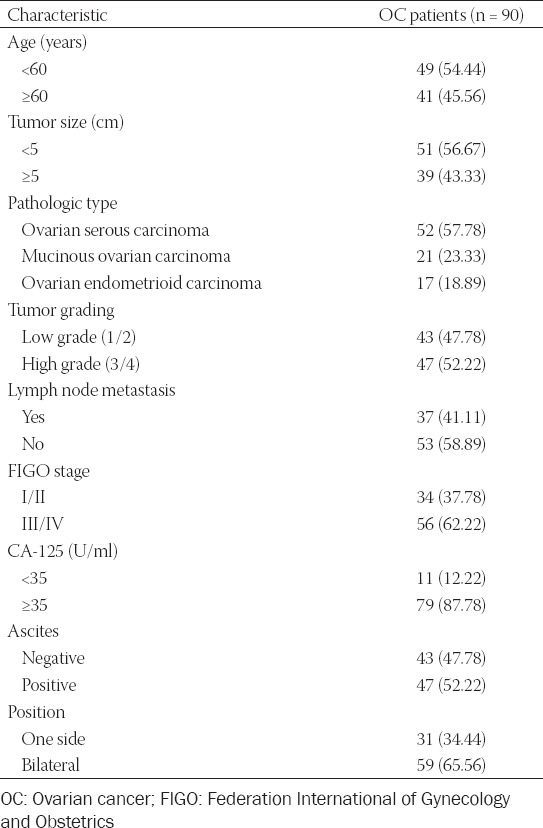
Baseline characteristics of enrolled OC patients

### Characteristics of exosomes

**FIGURE 2:**
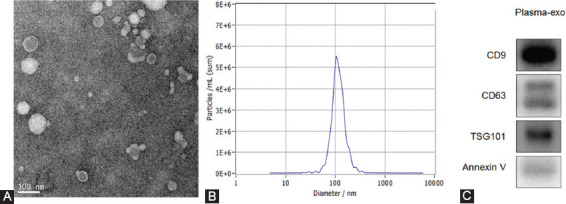
Patient exosome characterization (A) TEM images had a clear background and showed that the single exosome diameter was in the range of 100 nm and 200 nm; all the exosomes were clustered and connected. Exosome was encapsulated by holonomic lipid capsule and exhibited double disc-like vesicular structure; (B) Nanoparticle tracking analysis data showed that diameter for some particles was between 0 and 50 nm but their main distributional range was between 50 and 200 nm; the median value of the total particles was approximately 100 nm; (C) Western blotting showed positive expression for CD9, CD63, Tsg101, and Annexin V.

The purification and exosome integrity were verified by TEM, NTA, and western blot analysis. TEM images had a clear background and revealed that the single exosome diameter was between 100 nm and 200 nm and all the exosomes were clustered and connected. It was enclosed by a holonomic lipid capsule with a double disc-like vesicle structure (Figure 2A). The NTA data showed that a few particle diameters were between 0 and 50 nm and the main distribution was between 50 and 200 nm, the median value of the total particles was approximately 100 nm (Figure 2B). Western blot displayed that the expression of CD9, CD63, Tsg101, and Annexin V was all positive (Figure 2C).

### Correlation between plasma exosome-derived FATS levels and clinical-pathological parameters

**FIGURE 3:**
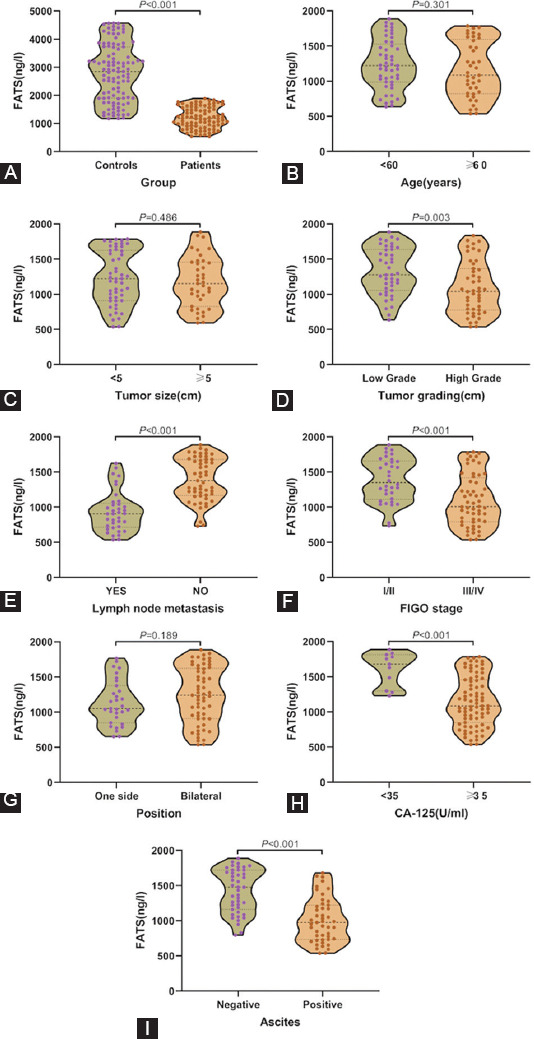
The levels of plasma exosome-derived fragile site-associated tumor suppressor (FATS) in ovarian cancer (OC) patients and healthy controls (A) The plasma exosome-derived FATS levels in OC patients and healthy controls; the plasma exosome-derived FATS levels in OC patients aged < 60 and >60 years (B), tumor size (C), grade (D), lymph node metastasis state (E), Federation International of Gynecology and Obstetrics stages (F), sides (G), CA-125 levels (H), and status of ascites (I).

**FIGURE 4:**
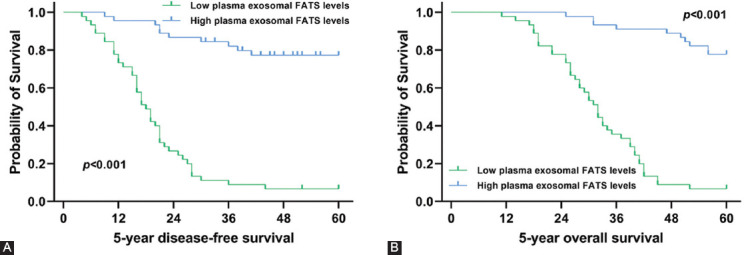
Association of plasma exosome-derived fragile site-associated tumor suppressor levels with 5-DFS and 5-OS in ovarian cancer (OC) patients. Survival curves for plasma exosome-derived FATS levels for prediction of 5-year DFS (A) and 5-year OS (B) in OC patients.

We compared the levels of plasma exosome-derived FATS between OC patients and healthy controls. The results showed that the exosome-derived FATS levels in OC patients were significantly down-regulated (*p* < 0.001; Figure 3A). We also evaluated the levels of plasma exosome-derived FATS in OC patients at different stages of the disease. The levels of plasma exosome-derived FATS were significantly higher in OC patients with lower grade (1/2) as compared to those with higher grade (3/4) (*p* = 0.003; Figure 3D). The levels of plasma exosome-derived FATS were significantly higher in OC patients with no lymph node metastases than those with lymph node metastases (*p* < 0.001; Figure 3E). The levels of plasma exosome-derived FATS were significantly higher in OC patients with FIGO Stages I/II as compared to Stages III/IV (*p* < 0.001; Figure 3F). The levels of plasma exosome-derived FATS were significantly higher in OC patients having CA-125 below 35 U/ml as compared to those with the value above 35 U/ml (*p* < 0.001; Figure 3H). The levels of plasma exosome-derived FATS were significantly higher in OC patients with no ascites as compared to those with ascites (*p* < 0.001; Figure 3I). However, there was no statistically significant difference among the patients of different ages, tumor diameters, and tumor positions (all *p* > 0.05; Figure 3B, 3C, 3G). Kaplan–Meier curves showed that both 5-OS and 5-DFS of OC patients with lower levels of plasma exosome-derived FATS were shorter as compared to those with higher levels (both *p* < 0.001; Figure 4A, B).

### The predictive ability of plasma exosome-derived FATS for 5-year survival of OC patients

Next, we evaluated the predictive ability of exosome-derived FATS for 5-year survival of OC patients (Table 2). The AUC for 5-DFS was 0.85 (95% CI: 0.76-0.91), with a sensitivity of 73.9 (95% CI: 61.9-83.7) and specificity of 81.0 (95% CI: 58.1-94.6). The cutoff value was 1277.41. The positive predictive value was 92.7 (95% CI: 82.4-98.0) and the likelihood ratio was 3.88 (95% CI: 1.6-9.5). The negative predictive value and likelihood ratio were 48.6 (95% CI: 31.4-66.0) and 0.32 (95% CI: 0.2-0.5), respectively.

**TABLE 2:**
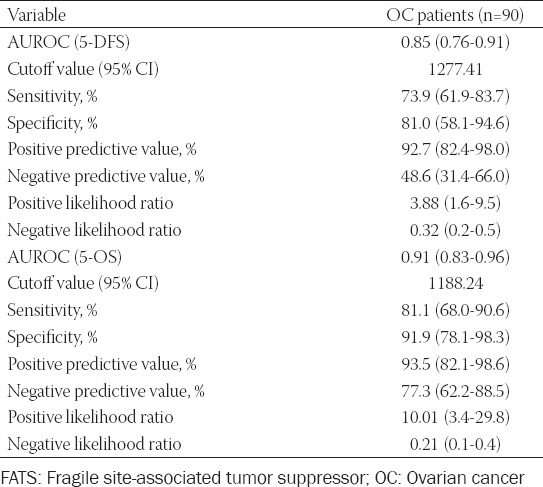
The predictive ability of plasma exosome-derived FATS for 5-year survival of patients with OC

The AUC for 5-OS was 0.91 (95% CI: 0.83-0.96), with a cutoff value of 1188.24. The positive predictive value and likelihood ratio were 93.5 (95% CI: 82.1-98.6) and 10.01 (95% CI: 3.4-29.8), respectively; while the negative predictive value and likelihood ratio were 77.3 (95% CI: 62.2-88.5) and 0.21 (95% CI: 0.1-0.4), respectively. The sensitivity and specificity were 81.1 (95% CI: 68.0-90.6) and 91.9 (95% CI: 78.1-98.3), respectively.

## DISCUSSION

Genomic instability is a common feature of tumor cells, characterized by abnormal DNA damage repair mechanisms and aberration of cell cycle regulation [21]. The occurrence and development of the tumor is the result of multigene and multistage synergy between genetic factors such as oncogene activation and tumor suppressor gene inactivation; it is affected by several environmental factors [22]. DNA damage is an important mechanism in the occurrence and development of malignant tumors. Fragile sites of tumor suppressor genes on chromosomes are highly sensitive to DNA damage. Reduced expression or deletion of these genes is highly correlated with the occurrence and development of tumors [23]. In recent years, evidence for fragile sites is accumulating [24]. Fragile sites are unstable regions in the normal genome; these include 39 rare fragile sites and 88 CFS. In normal cell culture, there are chromosome fragile sites. When DNA replication in cells is partially inhibited, a gap or a break region is formed during the metaphase in mitosis; this has been found in many cancers [25]. CFS locus genes are evolutionarily conserved and are closely related to tumorigenesis and development. Simultaneously, they may also participate in the regulation of signaling pathways in DNA damage response. At present, the development of tools for the identification of CFS locus genes, discovery and identification of new CFS genes, and investigating the relationship between CFS genes and carcinogenesis are challenging frontier topics for research. Recent studies show that the FATS gene is a potential candidate molecular marker which plays a key regulatory role in the early stages of tumorigenesis, and thus, could be used in molecular diagnoses and risk prediction for cancer. Therefore, it is of great significance for the study of tumorigenesis and development and can augment the development of new strategies for the prevention and treatment of cancers [12,13,26].

Song et al. have demonstrated the role of the FATS-p53 signaling cascade in the inhibition of pregnancy-related carcinogenesis and the potential application of FATS genotyping in breast cancer prevention [12]. Zhang et al. showed that the low expression of FATS is associated with breast cancer, and its expression has a good predictive value in the DFS of breast cancer patients [13]. Wu et al. validated that the expression of FATS protein is related to the occurrence and development of non-small cell lung cancer (NSCLC), and is an independent prognostic factor of NSCLC. The detection of FATS protein is expected to become a novel candidate biomarker for the evaluation of prognosis in patients with NSCLC [10]. However, the expression and biological roles, especially in exosomes, underlying FATS in OC remain unclear.

In this study, we have extracted exosomes from the plasma of OC patients. We have verified the purification and exosomal integrity using TEM, NTA, and western blotting. Next, we compared the levels of plasma exosome-derived FATS between OC patients and healthy controls. Compared with the healthy controls, exosome-derived FATS levels in OC patients were significantly downregulated. Next, we evaluated the levels of plasma exosome-derived FATS in OC patients at different stages of the disease. The levels of plasma exosome-derived FATS were significantly higher in OC patients with lower grade (1/2) as compared to those with higher grades (3/4). The levels of plasma exosome-derived FATS were significantly higher in OC patients with no lymph node metastases as compared to those with lymph node metastases. The levels of plasma exosome-derived FATS were significantly higher in OC patients with FIGO Stages I/II than Stages III/IV. The levels of plasma exosome-derived FATS were significantly higher in OC patients with CA-125 less than 35 U/ml as compared to those with values more than 35 U/ml. The levels of plasma exosome-derived FATS were significantly higher in OC patients with no ascites as compared to those with ascites. However, there were no statistically significant differences between patients with different ages, tumor diameters, and tumor positions. Among all enrolled OC patients, those with low plasma exosome-derived FATS levels had both shorter 5-DFS and 5-OS.

We also evaluated the prognostic value of plasma exosome-derived FATS levels for predicting the 5-year survival of OC patients through ROC curve analysis. The AUC of plasma exosome-derived FATS was 0.85 (95% CI: 0.76-0.91) for 5-DFS prediction in patients with OC, with a sensitivity of 73.9 (95% CI: 61.9-83.7) and a specificity of 81.0 (95% CI: 58.1-94.6). For OS prediction in OC patients, the AUC of plasma exosome-derived FATS was 0.91 (95% CI: 0.83-0.96), with a sensitivity of 81.1 (95% CI: 68.0-90.6) and a specificity of 91.9 (95% CI: 78.1-98.3). Thus, plasma exosome-derived FATS levels had good prediction performances for both 5-OS and 5-DFS of OC patients.

However, the study also has some limitations. First, although we had a larger study sample size for the evaluation of plasma exosome-derived FATS levels in OC patients, more patients from multiple centers need to be enrolled for validation of these results. Second, we did not assess the levels of plasma exosome-derived FATS in OC patients with different pathological types. Finally, we did not investigate the mechanisms of exosome-derived FATS in the occurrence and development of OC.

In summary, plasma exosome-derived FATS levels in OC patients were significantly downregulated. Low plasma exosome-derived FATS levels were closely related to the FIGO Stages III/IV, high grade, ascites, higher levels of CA-125, lymph node metastases, and prognoses of OC patients. These findings may facilitate the establishment of plasma exosome-derived FATS levels as a novel biomarker for OC prognosis.
